# Pigmented perivascular epithelioid cell tumor (PEComa) arising from kidney: A case report

**DOI:** 10.1097/MD.0000000000005248

**Published:** 2016-11-04

**Authors:** Hexi Du, Jun Zhou, Lingfan Xu, Cheng Yang, Li Zhang, Chaozhao Liang

**Affiliations:** Department of Urology, the First Affiliated Hospital of Anhui Medical University and Institute of Urology, Anhui Medical University, Hefei, Anhui, China.

**Keywords:** kidney, PEComa, pigmentation

## Abstract

**Introduction:**

Perivascular epithelioid cell tumor (PEComa) is a mesenchymal neoplasm composed of perivascular epithelioid cells with clear to eosinophilic cytoplasm. Pigmented PEComa arising from kidney is extraordinarily rare and sometimes can exhibit aggressive biological behavior.

**Case report:**

We present here a rare case of pigmented renal PEComa in a 46-year-old female. The patient had complained of lumbago complicated with nausea and vomiting for 2 weeks and therefore was referred to our department. An enhanced computed scan revealed a 4 × 3 × 3 cm round-like mass in the lower pole of right kidney with inhomogeneous enhancement. The tumor cells immunestained was positive for HMB-45, focally positive for c-Kit (CD117), and negative for vimentin, S-100, AE1/AE3, CK-7, CK-18, CD-10, RCC antigen, CgA, DOG-1, EMA, smooth muscle actin, and synaptophysin. We successfully performed 3-dimensional laparoscopic resection of the neoplasm, which was then diagnosed as pigmented PEComa by postoperative pathology. No further growing lesion or metastasis was observed during a 1-year follow-up.

**Conclusion:**

This case report shows that pigmented renal PEComa is often presented as a renal mass with nonspecific symptoms and imaging features. The gold diagnosis of renal pigmented PEComa is mainly based on the combination of histopathology and immunohistochemistry. Complete resection by 3-dimensional laparoscopic nephron-sparing surgery can be an effective therapeutic management.

## 1 Introduction

Perivascular epithelioid cell tumor (PEComa) is a mesenchymal tumor composed of perivascular epithelioid cells with immunohistochemical characteristics of smooth muscle and melanocytic markers.^[1]^ Although the morbidity of this tumor can happen with either sex, it has a predilection for women.^[2]^ Unlike typical angiomyolipoma, PEComa can display an aggressive clinical course with a 47% rate of disease progression.^[3]^ Pigmented PEComa of the kidney is extraordinarily rare that only few cases have been reported.^[2,4–8]^ As a variant of classic PEComa, most of these tumors were absent of tuberous sclerosis. Imaging features of pigmented renal PEComa is nonspecific, usually mimicking a renal cell carcinoma, and the biologic behavior is undetermined. Here, we present a rare case of a female patient diagnosed as pigmented right renal PEComa with the aim to share the experience regarding diagnosis and therapy.

## 2 Consent

Informed consent was signed by the patient for the publication of this report and related images.

## 3 Case report

A 46-year-old woman complained of right flank pain with nausea and vomiting for 2 weeks. She was referred to our department for further examination and treatment after an ultrasonographic suggestion of hypo-echoic renal lesion in the local hospital. By inquiring the case history, we learnt that she suffered from diabetes for about 5 years. Apart from Cesarean section that was performed 22 years before, no other surgical history was present. In addition, an abdominal physical examination disclosed no positive findings.

**Figure 1:**
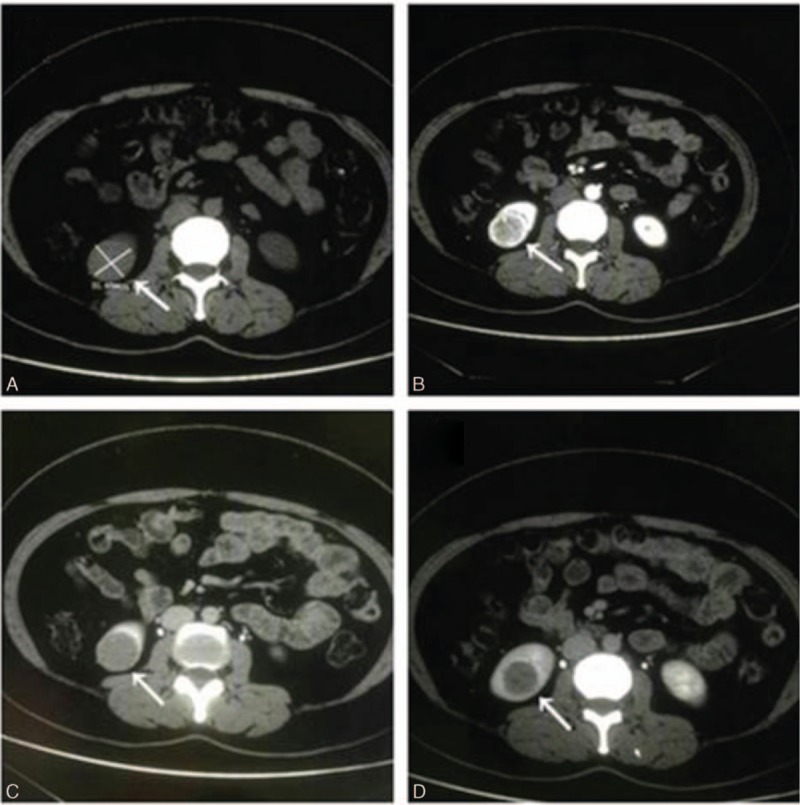
(A) Plain CT scan revealed a 4 × 3 × 3 cm well-demarcated mass on the lower pole of right kidney (white arrow). The tumor was enhanced nonuniformly during arterial phase (B), whereas no enhancement was shown during venous phase (C) and renal parenchymal phase (D). CT = computed tomography.

After admission, we conducted a routine form of laboratory test and found all data were within normal limits except for a remarkably increased level of fasting blood glucose (11.79 mmol/L). Abdominal enhanced computer tomography (CT) showed a 4 × 3 × 3 cm round-like, slightly hyperdense mass lesion in the lower pole of right kidney with inhomogeneous enhancement (Fig. 1). There was no sign of organ infiltration and distant metastasis. No abnormalities were revealed from electrocardiogram examination as well as chest x-ray. Thus, taken together with the result of physical examination, imaging findings, and related laboratory test, a clinical diagnosis of right renal tumor was made. Preoperatively, the patient's blood glucose was controlled to normal level.

**Figure 2:**
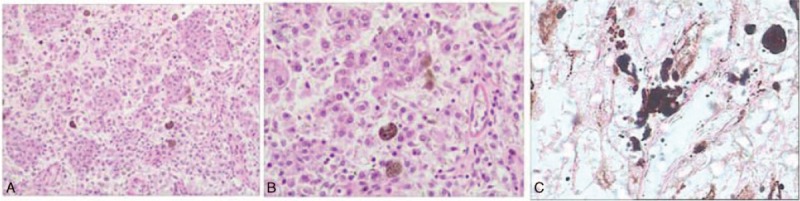
The histopathologic examination showed abundant polygonal epithelioid cells with dense eosinophilic or clear cytoplasm and round nuclei (H&E × 100, A; ×200, B). The cytoplasmic black pigment was highlighted by Fontana–Masson stain (×200, C). H&E = hematoxylin and eosin.

**Figure 3:**
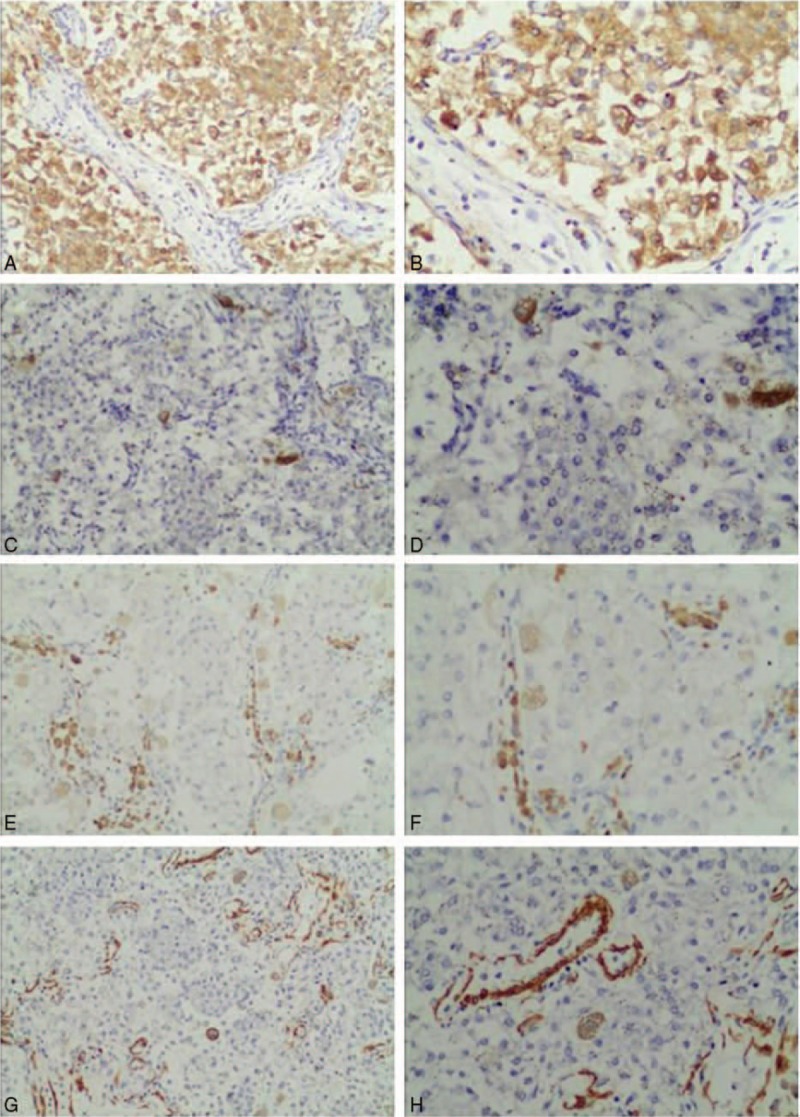
Immunohistochemical appearance demonstrated that tumor cells were positive for HMB45 (×100, A; ×200, B), focally positive for c-Kit (×100, C; ×200, D), and negative for CK-18 (×100, E; ×200, F), SMA (×100, G; ×200, H). SMA = smooth muscle actin.

According to the tumor size and anatomic characteristics, we finally performed 3-dimensional (3D) retroperitoneoscopic nephron-sparing surgery and resected the mass lesion completely. Operatively, it was easy to note an external tumor located at the lower level of the kidney. The operation was a success with a warm ischemia time of 26 minutes. No intraoperative transfusion or complication occurred during the surgery. On gross examination after the surgery, the thin capsule of tumor specimen was found to be intact. Microscopically, the tumor was composed of polygonal tumor cells with dense eosinophilic or clear cytoplasm and round to oval nuclei (Fig. 2). Melanin pigment could be found reacting strongly with Fontana–Masson stain in their cytoplasm, and the nuclear pleomorphism was hardly to be seen in the nucleus. By immunohistochemical examination (Fig. 3), the tumor was strongly positive for HMB-45, focally positive for c-Kit (CD117), and negative for vimentin, S-100, AE1/AE3, CK-7, CK-18, CD-10, RCC antigen, CgA, DOG-1, EMA, smooth muscle actin (SMA), and synaptophysin. According to these findings, the pathological analysis suggested pigmented right renal PEComa.

During the postoperative period, the patient had an uneventful course and was discharged after 6 days. Up to the nearest follow-up (1 year after operation), the woman was without any discomfort and free of recurrence detected by radiology.

## 4 Discussion

Perivascular epithelioid cell tumor occurs in a variety of sites, especially common in liver, prostate, and uterine cervix.^[9–11]^ The PEComa family of tumors includes angiomyolipoma, lymphangioleiomyomatosis, clear cell sugar tumor of the lung, clear cell myomelanocytic tumor of the falciform ligament/ligamentum teres, and rare clear cell tumors of other anatomical sites (PEComas-NOS).^[12]^ Pigmented PEComa of the kidney was unusual and first reported by Fujimoto et al.^[13]^ It manifests in older patients, with an average age of 44 and a female predominance.^[2,5]^

The presenting symptoms of pigmented renal PEComa are usually nonspecific, including back ache, febrile, hematuria.^[6,7]^ With respect to laboratory tests, the main results are mostly in normal range. The presence of pigmented renal PEComa in CT images are also lack of characteristic signs and can often appear as solitary solid mass with inhomogeneous enhancement. Since this type of tumor is without macroscopic fat that we commonly see in typical angiomyolipoma, it can easily be mistaken for renal carcinoma by radiographic evaluation.^[14]^

Diagnosis of pigmented renal PEComa is heavily dependent on the combination of histopathology and immunohistochemistry. On microscopic examination, the tumor is characteristically composed purely of epitheloid cells and absent of fat cells and the blood vessels.^[6]^ Unlike the typical PEComa, which co-expresses melanocytic markers (HMB-45, HMB50, Melan-A) and smooth muscle markers (smooth muscle actin, muscle specific actin, calponin), the pigmented PEComa of kidney distinctively exhibits immunohistochemical reaction with HMB-45 but hardly with muscle markers. Vimentin, S-100, CD68, and c-Kit have also been detected in some of the pigmented renal PEComa.^[7,8]^ In our case, a panel of antibodies including: epithelial membrane antigen, cytokeratin antigens (AE1/AE3, CK7, CK18), mesenchymal antigens (SMA, vimentin, c-Kit, DOG-1), carcinoembryonic antigen (RCC antigen), neuroendocrine antigens (S-100, CgA, synaptophysin), adenoid antigen (CD-10), and HMB-45 were used to identify the immunoprofile of the tumor. What should be pointed out is that, to our knowledge, the pigmented PEComa of kidney in this article is the second reported case of c-Kit positivity.

The major differential diagnosis of pigmented PEComa includes malignant melanoma, pigmented clear cell renal cell carcinoma, composite paraganglioma, and melanotic Xp11 translocation renal cancer. These tumors each is to be separated from pigmented PEComa by their distinctive features. For example, the melanotic Xp11 translocation renal cancer has TFE3 rearrangement, which is not observed in the pigmented PEComa.^[15]^

Complete surgical resection of the tumor is the suggested mainstay treatment of pigmented renal PEComas. As in this case, we selected the retroperitoneal 3D laparoscopic partial nephrectomy in the management of the mass, which was extremely beneficial to the preservation of renal function. Our result indicated that surgery alone can be an effective treatment method for the PEComa. However, for those with large, unresectable, malignant, or metastatic tumor, adjuvant chemotherapy and radiotherapy may associate with a prolongation of survival.^[16]^ The mechanism of chemotherapy is related to the inhibition of mTOR (mammalian target of rapamycin) pathway, which plays a key part in the regulation of cell growth and proliferation.^[17]^

As certain aggressive cases have been reported,^[12,13]^ the prognosis of pigmented PEComa is not optimistic. There are no reliable morphologic criteria of malignancy other than widespread metastasis. However, Nese et al^[18]^ held that necrosis, diameter >7 cm, presence of tuberous sclerosis, extrarenal extension or renal vein invasion, carcinoma-like growth pattern were the 5 negative prognostic factors associated with disease progression. Therefore, careful, long-term follow-up is needed for patients with pigmented PEComa of the kidney.

## 5 Conclusions

In the present case, we demonstrate a rare disease of pigmented renal PEComa. The gold diagnosis of renal pigmented PEComa is mainly based on the combination of histopathology and immunohistochemistry. Three-dimensional laparoscopic nephron-sparing surgery can be used feasibly and effectively for treatment of this type of tumor. Meanwhile, to evaluate its prognostic implications, a long-term follow-up and a comprehensive study of more pigmented PEComa patients are needed.

## References

[1] Hasan H Howard AF Alassiri AH PEComa of the terminal ileum mesentery as a secondary tumour in an adult survivor of embryonal rhabdomyosarcoma. *Curr Oncol* 2015; 22:e383–386. 26628881 10.3747/co.22.2265 PMC4608414

[2] Chang H Jung W Kang Y Pigmented perivascular epithelioid cell tumor (PEComa) of the kidney: a case report and review of the literature. *Koreea J Pathol* 2012; 46:499–502. 10.4132/KoreanJPathol.2012.46.5.499 PMC3490117 23136579

[3] Shrewsberry AB Sica GL Osunkoya AO Epithelioid PEComa (epithelioid angiomyolipoma) of the kidney: a rare tumor subtype for patients presenting with an enhancing renal mass. *Can J Urol* 2013; 20:6643–6645. 23433138

[4] Fukunaga M Harada T Pigmented perivascular epithelioid cell tumor of the kidney. *Arch Pathol Lab Med* 2009; 133:1981–1984. 19961256 10.5858/133.12.1981

[5] Reddy R Lewin JR Shenpy V Pigmented epithelioid angiomyolipoma of the kidney. *J Miss State Med Assoc* 2015; 56:92–94. 26118214

[6] Rasalkar DD Chu WC Chan AW Malignant pigmented clear cell epithelioid cell tumor (PEComa) in an adolescent boy with widespread metastases: a rare entity in this age group. *Pediatr Radiol* 2011; 41:1587–1590. 21597905 10.1007/s00247-011-2125-0

[7] Yu W Fraser RB Gaskin DA C-Kit-positive metastatic malignant pigmented clear-cell epithelioid tumor arising from the kidney in a child without tuberous sclerosis. *Ann Diagn Pathol* 2005; 9:330–334. 16308163 10.1016/j.anndiagpath.2005.04.003

[8] Ribalta T Lloreta J Munné A Malignant pigmented clear cell epithelioid tumor of the kidney: clear cell (“sugar”) tumor versus malignant melanoma. *Hum Pathol* 2000; 31:516–519. 10821501 10.1053/hp.2000.6717

[9] Ameurtesse H Chbani L Bennani A Primary perivascular epithelioid cell tumor of the liver: new case report and literature review. *Diagn Pathol* 2014; 9:149. 25034830 10.1186/1746-1596-9-149 PMC4223599

[10] Eken A Saglican Y Primary perivascular epithelioid cell tumour (PEComa) of the prostate. *Can Urol Assoc J* 2014; 8:E455–E457. 25024807 10.5489/cuaj.1752 PMC4081268

[11] Tajima S Koda K Perivascular epithelioid cell tumor of the uterine cervix identified on a conventional cervical smear. *Diagn Cytopathol* 2015; 43:1011–1016. 26390283 10.1002/dc.23369

[12] Patra S Vij M Kota V Pigmented perivascular epithelioid cell tumor of the liver: report of a rare case with brief review of literature. *J Cancer Res Ther* 2013; 9:305–307. 23771383 10.4103/0973-1482.113401

[13] Fujimoto H Chitose K Tobisu K Solitary renal melanoma? A case with long survival after initial treatment. *J Urol* 1995; 153:1887–1889. 7752341

[14] Ryan MJ Francis IR Cohan RH Imaging appearance of renal epithelioid angiomyolipomas. *J Comput Assist Tomogr* 2013; 37:957–961. 24270119 10.1097/RCT.0b013e3182a77674

[15] Ritterhouse LL Cykowski MD Hassell LA Melanotic Xp11 translocation renal cancer: report of a case with a unique intratumoral sarcoid-like reaction. *Diagn Pathol* 2014; 9:81. 24735727 10.1186/1746-1596-9-81 PMC4003493

[16] Zhao Y Bui MM Spiess PE Sclerosing PEComa of the kidney: clinicopathologic analysis of 2 cases and review of the literature. *Clin Genitourin Cancer* 2014; 12:e229–e232. 25044147 10.1016/j.clgc.2014.04.009

[17] Martignoni G Pea M Zampini C PEComas of the kidney and of the genitourinary tract. *Semin Diagn Pathol* 2015; 32:140–159. 25804448 10.1053/j.semdp.2015.02.006

[18] Nese N Martignoni G Fletcher CD Pure epithelioid PEComas (so-called epithelioid angiomyolipoma) of the kidney: a clinicopathologic study of 41 cases: detailed assessment of morphology and risk stratification. *Am J Surg Pathol* 2011; 35:161–176. 21263237 10.1097/PAS.0b013e318206f2a9

